# Supplementary material for: Synthesis of Sodium Cobalt Fluoride/Reduced Graphene Oxide (NaCoF3/rGO) Nanocomposites and Investigation of Their Electrochemical Properties as Cathodes for Li-Ion Batteries
Source: Materials (Basel). 2021 Jan 24;14(3):547. doi: 10.3390/ma14030547 (PMC7865758; doi:10.3390/ma14030547)
Supplement: Supplementary file 1 [file materials-14-00547-s001.pdf]

materials

Supplementary Materials

Synthesis of Sodium Cobalt Fluoride/Reduced Graphene Oxide ($NaCoF_3$/rGO) Nanocomposites and Investigation of Their Electrochemical Properties as Cathodes for Li-Ion Batteries

Jiwoong Oh [1,†], Jooyoung Jang [1,†], Eunho Lim [2,*], Changshin Jo [1,*] and Jinyoung Chun [3,*]

1 School of Chemical Engineering and Materials Science, Chung-Ang University (CAU), 84 Heukseok-ro, Dongjakgu, Seoul 06974, Korea; shanall@cau.ac.kr (J.O.); wndud2362@cau.ac.kr (J.J.)
2 Chemical & Process Technology Division, Korea Research Institute of Chemical Technology (KRICT), 141 Gajeongro, Daejeon 34114, Korea
3 Energy and Environmental Division, Korea Institute of Ceramic Engineering and Technology (KICET), Jinju, Gyeongnam 52851, Korea
* Correspondence: eunholim@krict.re.kr (E.L.); changshin@cau.ac.kr (C.J.); jchun@kicet.re.kr (J.C.)
† These authors contributed equally to this work.

Citation: Oh, J.; Jang, J.; Lim, E.; Jo, C.; Chun, J. Synthesis of Sodium Cobalt Fluoride/Reduced Graphene Oxide (NaCoF3/rGO) Nanocomposites and Investigation of Their Electrochemical Properties as Cathodes for Li-Ion Batteries. *Materials* **2021**, *14*, 547. https://doi.org/10.3390/ma14030547

Received: 8 January 2021
Accepted: 21 January 2021
Published: 24 January 2021

Publisher's Note: MDPI stays neutral with regard to jurisdictional claims in published maps and institutional affiliations.

Copyright: © 2021 by the authors. Submitted for possible open access publication under the terms and conditions of the Creative Commons Attribution (CC BY) license (http://creativecommons.org/licenses/by/4.0/).

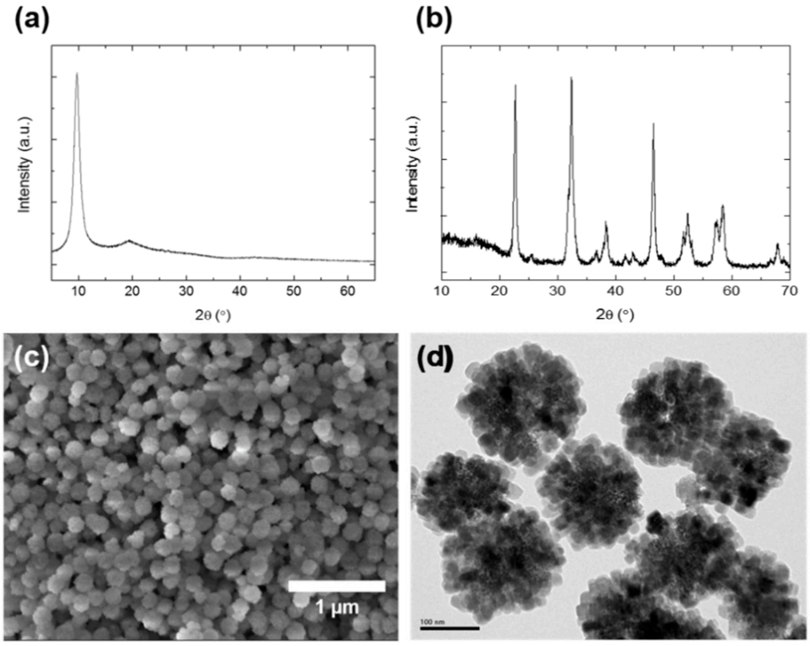

Figure S1. (**a**) XRD pattern of GO, (**b**) XRD pattern, (**c**) SEM image, and (**d**) TEM image of NCF NCs.

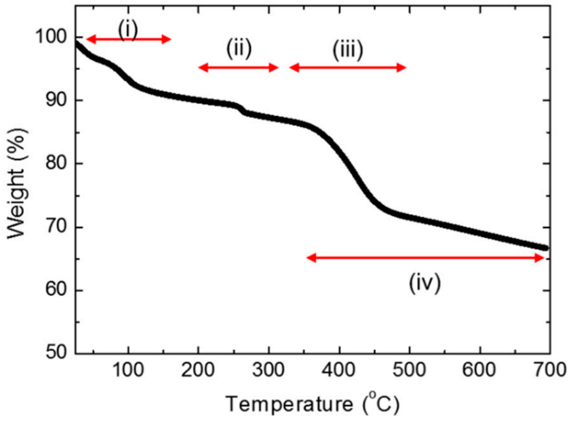

Figure S2. Thermogravimetric analysis of NCF/rGO nanocomposites.

Figure S2 exhibited thermogravimetric analysis (TGA). Mass loss of (i) means the removal of impurities such as attached moisture (−9%) and slightly mass loss of (ii) exhibited decomposition of surface coupling with citrate, acetate moieties. Mass loss of (iii) reflects the decomposition of rGO. Considering the presence of a small amount of oxygen functional groups, it is judged that the results roughly agree with the results of elemental analysis (carbon content: 13.8 wt%). Moreover, mass loss of (iv) exhibited that it is the mass reduction in the process of decomposition ($NaCoF_3 \rightarrow NaF + Co_3O_4$). To sum up all the information, excluding impurities such as moisture, the weight ratio of NCF:rGO in the composite was estimated to be 85:15.

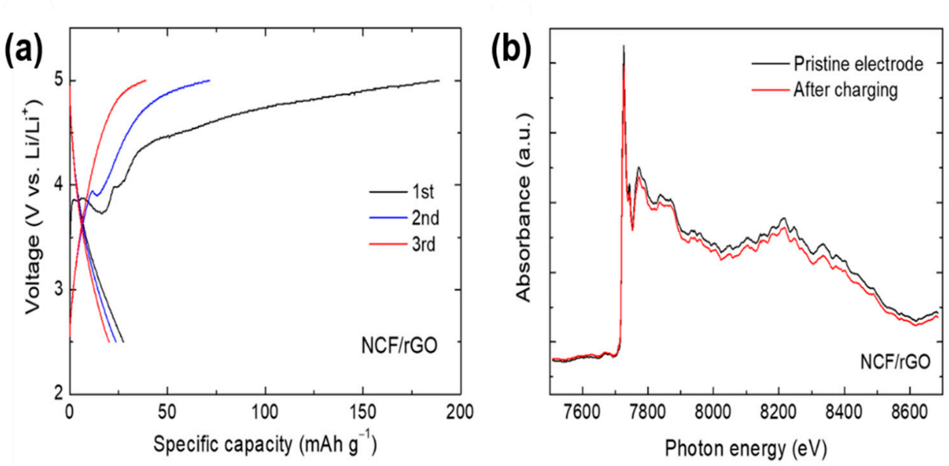

Figure S3. (**a**) Galvanostatic charge/discharge curves of the 1st, 2nd, and 3rd cycles of NCF/rGO at 0.1C, (**b**) X-ray absorption near edge structure (XANES) measurements of pristine and charged electrode.
